# Endothelial dysfunction and carotid atherosclerosis in Malawian adults: A cross-sectional study

**DOI:** 10.1016/j.ensci.2020.100252

**Published:** 2020-06-28

**Authors:** Joseph Kamtchum-Tatuene, Gloria Mwangalika Kachingwe, Henry C. Mwandumba, Tom Solomon, Laura A. Benjamin

**Affiliations:** a Malawi-Liverpool-Wellcome Trust Clinical Research Programme, University of Malawi College of Medicine, Blantyre, Malawi; b Neuroscience and Mental Health Institute, Faculty of Medicine and Dentistry, University of Alberta, Edmonton, Alberta, Canada; c Department of Clinical Sciences, Liverpool School of Tropical Medicine, Liverpool, United Kingdom; d Institute of Infection and Global Health, University of Liverpool, Liverpool, United Kingdom; e Institute of Neurology, University College of London, London, United Kingdom

**Keywords:** Endothelial dysfunction, HIV infection, Africa, Stroke, Atherosclerosis, Carotid intima-media thickness, cIMT, Carotid intima-media thickness, CV, Coefficient of variability, ELISA, Enzyme-linked immunosorbent assay, HIV, Human immunodeficiency virus, ICAM-1, Intercellular adhesion molecule 1, PAI-1, Plasminogen activator inhibitor 1, sTM, soluble thrombomodulin, VEGF, Vascular endothelial growth factor

## Abstract

**Background and objective:**

In sub-Saharan Africa, data on prevalence, risk factors and pathobiology of carotid atherosclerosis are scarce. We aimed to investigate the relationship between biomarkers of endothelial dysfunction and carotid atherosclerosis.

**Methods:**

Carotid ultrasound was performed in 66 patients. Plasma concentration of ICAM-1, PAI-1, VEGF, and soluble thrombomodulin were measured by ELISA. A univariable logistic regression analysis was performed to study the relationship between carotid atherosclerosis, biomarkers of endothelial dysfunction, and various demographic and clinical parameters of the participants.

**Results:**

The mean age of the participants was 58.7 years (95% CI: 54.4–63.1). Carotid atherosclerosis was diagnosed in 39.4% (95% CI: 27.6–52.2). In the univariable logistic regression, the following factors were associated with carotid atherosclerosis: age > 45 years (OR = 12.0, 95% CI: 1.4–98.8, *p* = .02), hypertension (OR = 3.8, 95% CI: 1.2–12.1, p = .02), and high-level of soluble thrombomodulin (OR = 3.4, 95% CI: 1.2–10.0, p = .02).

**Conclusions:**

There is an association between high levels of soluble thrombomodulin and carotid atherosclerosis in Malawian adults. Further studies with a larger sample size are needed to confirm our findings in other African populations.

## Highlights

• High levels of soluble thrombomodulin are associated with carotid atherosclerosis.
• Age > 45 years and hypertension are also associated with carotid atherosclerosis.
• Mannheim consensus criteria for assessing carotid intima-media thickness and plaque.

## 1 Introduction

Several studies carried out in Western countries have established that endothelial dysfunction, triggered by various cardiovascular risk factors, is the primum movens of the arterial remodelling process leading to atherosclerosis and then atherothrombotic events [1,2]. In sub-Saharan Africa, data on prevalence, risk factors and pathobiology of carotid atherosclerosis are scarce. To address this knowledge gap, we have conducted several prospective studies in Malawi, a country with >10% prevalence of HIV [[3], [4], [5], [6]]. In one study, we showed that endothelial dysfunction persists despite antiretroviral therapy and could, therefore, contribute to the development of atherosclerosis [5]. In a subsequent study, we reported a 39.4% prevalence of carotid atherosclerosis in a sample of 66 patients presenting with an acute stroke-like syndrome [7]. Plasma samples were also collected from the patients to investigate the relationship between biomarkers of endothelial dysfunction and carotid atherosclerosis. Here, we present and discuss the findings of these complimentary analyses.

## 2 Methods

### 2.1 Selection of patients and ultrasound assessment

Participants were recruited consecutively from April to August 2017 at the Queen Elizabeth Central Hospital which is the largest referral hospital in Malawi. The presence of any cardiovascular risk factor was recorded according to previously reported definitions [7].

Carotid ultrasound examination was performed on the day of enrolment (JKT) using the CX50 portable ultrasound device (Philips, Amsterdam, NL). Details of the examination protocol and criteria for the interpretation of findings were reported previously [7]. Briefly, the examination comprised a semi-automated assessment of the carotid intima-media thickness (cIMT) and a grading of any existing stenosis. Carotid atherosclerosis was defined as abnormal intima-media thickness or presence of carotid plaque with or without stenosis.

In order to define abnormal cIMT, the following age-specific cut-offs were applied: 0.60 mm (30–39 years old), 0.65 mm (40–49 years old), 0.70 mm (50–59 years old), 0.75 mm (60–69 years old), and 0.80 mm (≥70 years old). In the absence of reference values specific to African populations, the reference cut-off in each age category was chosen close to the upper limit of the 95% confidence interval of the mean cIMT for participants without cardiovascular risk factors enrolled in the PARC study (Paroi Artérielle et Risque Cardiovasculaire) [8]. Results of the PARC study suggest that the normal aging of the arterial vessel wall is associated with intima-media thickening by approximately 0.5 mm per decade. In patients with cardiovascular risk factors, the cIMT is typically higher that the indicated cut-off in each age category and is dependent on the number of cardiovascular risk factors as illustrated in the Mannheim consensus guidelines [9].

### 2.2 Measurement of biomarkers of endothelial dysfunction

We used ELISA kits from Abcam ® (Abcam plc, Cambridge, United Kingdom) to measure the plasma concentrations of biomarkers of endothelial activation (intercellular adhesion molecule, ICAM-1), endothelial damage (plasminogen activator inhibitor 1, PAI-1), angiogenesis (vascular endothelial growth factor, VEGF), and coagulopathy (soluble thrombomodulin, sTM). SimpleStep® ELISA kits were used for ICAM-1, PAI-1, and sTM while the standard kit was used for VEGF. All samples were run in duplicates and samples with an individual coefficient of variability (CV) greater than 25% were retested in duplicates.

All the ELISA tests were performed according to the manufacturer’s instructions with exception of the washing steps that were performed with an automated washer (ASYS Atlantis®, Biochrom Ltd., Cambridge, UK) using a mixture of phosphate buffer saline and Tween 20 (0.05% *v*/v). The plates were read at 450 nm using an automated microplate reader (EZ Read®, Biochrom Ltd., Cambridge, UK). The standard curves were built using 4-parameter logistic regression. The mean intra-assay CV for ICAM-1, PAI-1, VEGF, and sTM was 3.3%, 3.2%, 8.0%, and 3.1% respectively (accepted value <10%). The mean inter-assay CV for ICAM-1, PAI-1, VEGF, and sTM was 4.1%, 4.1%, 9.0%, and 2.8% respectively (accepted value <15%).

### 2.3 Statistical analysis

Participants' demographic and clinical parameters were summarized as proportions and means with 95% confidence interval for categorical and numerical variables, respectively. The relationship between plasma levels of biomarkers of endothelial dysfunction and cIMT was explored using the Spearman rank correlation test.

A univariable logistic regression analysis was performed to study the relationship between carotid atherosclerosis (dependent variable) and various demographic and clinical parameters of the participants. Following the approach in our previous publications [5,6], age was dichotomized at 45 years while biomarkers were dichotomized at the third tertile of the distribution of plasma concentration values (low versus high level). A multivariable analysis was not performed due to the small sample size. All statistical tests were two-tailed and statistical significance was defined as *p* ≤ .05. Analyses were performed using the software STATA (version 13, StataCorp, College Station, TX, USA).

## 3 Results

The mean age of the participants was 58.7 years (95% CI: 54.4–63.1). There were 22 HIV-positive patients and 77.3% were on antiretroviral therapy. HIV+ cases were younger (mean age 43.6 versus 66.3, *p* < .001) and their mean CD4+ T cell count was 223/mm^3^. There was no difference in the levels of biomarkers between HIV-positive and HIV-negative patients. The baseline characteristics of the participants are summarized in Table 1.

**Table 1:** Participants' baseline characteristics.

Characteristics^a^	Carotid atherosclerosis	
	Yes (*n* = 26)	No (*n* = 40)
Age (years)	63.0 (57.7–68.2)	56.1 (49.7–62.4)
Age > 45 years	25 (96.2)	27 (67.5)
Men	13 (50.0)	15 (37.5)
HIV positive	7 (27.0)	15 (37.5)
On ART	7 (26.9)	10 (25.0)
Smoking	3 (11.5)	5 (12.5)
Alcohol consumption	4 (15.4)	9 (22.5)
Hypercholesterolemia	3 (11.5)	0 (0.0)
Hypertension	21 (80.8)	21 (52.5)
Diabetes mellitus	5 (19.2)	6 (15.0)
Waist-hip ratio	0.9 (0.9–1.0)	0.9 (0.8–0.9)
CD4+ count	472.3 (377.7–566.9)	421.2 (320.2–522.2)
ICAM-1 (ng/mL)	377.4 (244.2–510.6)	404.7 (268.4–540.9)
PAI-1 (ng/mL)	43.0 (26.8–59.2)	49.3 (31.0–67.5)
sTM (ng/mL)	8.1 (5.6–10.6)	6.2 (5.1–7.4)
VEGF (pg/mL)	12.8 (0.0–26.7)	4.4 (0.9–8.0)

a Data are presented as N (%) or mean (95%CI).

There was no correlation between the plasma concentration of the biomarkers of endothelial dysfunction and the value of cIMT (Spearman rank correlation coefficient of −0.24, −0.03, 0.11, 0.11 for ICAM-1, PAI-1, sTM, and VEGF, respectively).

In the univariable logistic regression (Table 2), the following factors were associated with carotid atherosclerosis: age > 45 years (OR = 12.0, 95% CI: 1.4–98.8, *p* = .02), hypertension (OR = 3.8, 95% CI: 1.2–12.1, p = .02), and high-level of soluble thrombomodulin (OR = 3.4, 95% CI: 1.2–10.0, p = .02).

**Table 2:** Univariable analysis of factors associated with carotid atherosclerosis.

Characteristic^a^	Univariable analysis		
	OR	95% CI	p
HIV infection	0.6	0.2–1.8	0.38
Age > 45	12.0	1.4–98.8	0.02
Male	1.6	0.6–4.5	0.32
Hypertension	3.8	1.2–12.1	0.02
Diabetes mellitus	1.3	0.4–5.0	0.65
Smoking	0.9	0.2–4.2	0.91
Waist-hip ratio (*3rd tertile)*	1.9	0.7–5.5	0.22
ART started	1.1	0.4–3.4	0.86
High level of ICAM-1	0.8	0.3–2.4	0.72
High level of PAI-1	1.1	0.4–3.1	0.86
High level of sTM	3.4	1.2–10.0	0.02
High level of VEGF	1.8	0.6–5.5	0.32

a There were 3 patients with hypercholesterolemia and they all had carotid atherosclerosis.

## 4 Discussion

This study is the first to investigate the relationship between biomarkers of endothelial dysfunction and carotid atherosclerosis in sub-Saharan Africa. The association between high levels of soluble thrombomodulin and carotid atherosclerosis is in line with findings in Western populations [10,11]. Soluble thrombomodulin is an endothelial membrane glycoprotein also known as CD141. It is a marker of endothelial injury and higher plasma levels have been associated with other risk factors of atherosclerosis, notably age and hypertension [12]. In this study, patients with carotid atherosclerosis had a higher mean age and a higher prevalence of hypertension, which could contribute to the association between high levels of soluble thrombomodulin and carotid atherosclerosis. Due to the small sample size of this exploratory study, we could not reliably assess the independence of the association in a multivariable analysis. Further studies with a larger sample size are, therefore, needed to confirm our findings in African populations and investigate the performance of biomarkers of endothelial dysfunction as predictors of cardiovascular events. The small sample size likely also explains why our analysis did not show an association between carotid atherosclerosis and other established drivers of endothelial dysfunction and atherosclerosis, notably smoking, diabetes mellitus, adiposity, and HIV infection [[13], [14], [15], [16]]. Specifically, for HIV-positive patients who are younger, longitudinal studies are needed to investigate if the persistence of HIV-induced endothelial dysfunction has a significant and independent impact on the incidence of carotid atherosclerosis and whether antiretroviral or anti-inflammatory drugs are useful to prevent its progression and cerebrovascular consequences. Such studies should ideally be performed on large sample size populations and follow international consensus recommendations for the assessment of subclinical and clinical atherosclerosis in order to avoid generating more heterogeneous or contradictory results [17].

## Statement of ethics

This work was conducted as part of the study of Biomarkers of Stroke and Arterial diseases in Malawian Adults (BIOSTATA). The study protocol was approved by the Human Research Ethics Committees of the Liverpool School of Tropical Medicine (16–035) and the University Of Malawi College Of Medicine (P.06/16/1974). A signed informed consent was obtained from all the patients enrolled or their next of kin. The research was conducted in accordance with the World Medical Association Declaration of Helsinki.

## Funding sources

This research was supported by the 10.13039/100010269Wellcome Trust [grant number 106829/Z/15/Z to JK-T].

The funder had no role in study design; data collection, analysis and interpretation; writing of the report; and decision to submit the article for publication.

## Declaration of Competing Interest

None.
